# Multifocal Brain Abscesses Due to Streptococcus intermedius

**DOI:** 10.7759/cureus.32797

**Published:** 2022-12-21

**Authors:** Raul D Isern, Stephen Toth, Mikhail Goldfarb, Faran Ahmad

**Affiliations:** 1 Division of Infectious Diseases, Creighton University School of Medicine, Omaha, USA; 2 Division of Internal Medicine, Creighton University School of Medicine, Omaha, USA; 3 Division of Critical Care Medicine, Creighton University School of Medicine, Omaha, USA

**Keywords:** streptococcus anginosus group, brain biopsy, brain abscess, multifocal brain abscesses, streptococcus intermedius

## Abstract

Brain abscess is a life-threatening illness that occurs when an intracerebral infection leads to cerebritis and subsequent pus formation within a well-vascularized capsule. While streptococci (aerobic, anaerobic, and microaerophilic) are the most common bacteria isolated, its presentation as multifocal brain abscesses is rarely described. In this report, we describe a 43-year-old male patient who presented to the emergency department due to progressive lethargy and low-grade fever of seven days worsening. Upon further evaluation, the patient was found to have multiple brain abscesses secondary to *Streptococcus intermedius,* confirmed by the culture of stereotactic aspiration of brain collection. This case underlines the importance of considering *Streptococcus intermedius *as a cause of multifocal brain abscesses.

## Introduction

Brain abscess is a localized intracerebral infection that has capsulized into a collection of pus. Studies have shown that brain abscess is estimated to occur in 0.4-0.9 cases per 100,000 population [1]. Incidence has also been described to be as high as 8% of intracranial masses in developing countries and 1%-2% of cases in Western countries [2]. Higher rates exist in immunocompromised patients. Several predisposing conditions have been described, including otitis media, sinusitis, dental infection, or immunosuppression. The clinical course ranges from indolent to fulminant [2]. The size and location of the abscess play a role in clinical presentation. Headache is the most common presenting symptom. The majority of brain abscesses are unicentric and result from peri-cranial infection [3].

## Case presentation

A 43-year-old male presented to the emergency department with somnolence, confusion, and weakness of two days that is worsening. Upon presentation, the patient reported associated fever, fatigue, sore throat, and “flu-like symptoms” of seven-day duration, with worsening in the past two days. He was unable to provide a comprehensive history of the present illness due to associated lethargy and somnolence. His family was at bedside and reported that two weeks prior, he went swimming and water tubing in the Platte River, a freshwater river. They also reported that two weeks prior, he had a sore throat, which prompted the use of cefdinir 400 mg three times a day (TID) for several days due to a concern for streptococcal pharyngitis. He had a cat at home for which he changed the cat litter routinely. The patient had no history of drug use.

**Figure 1:**
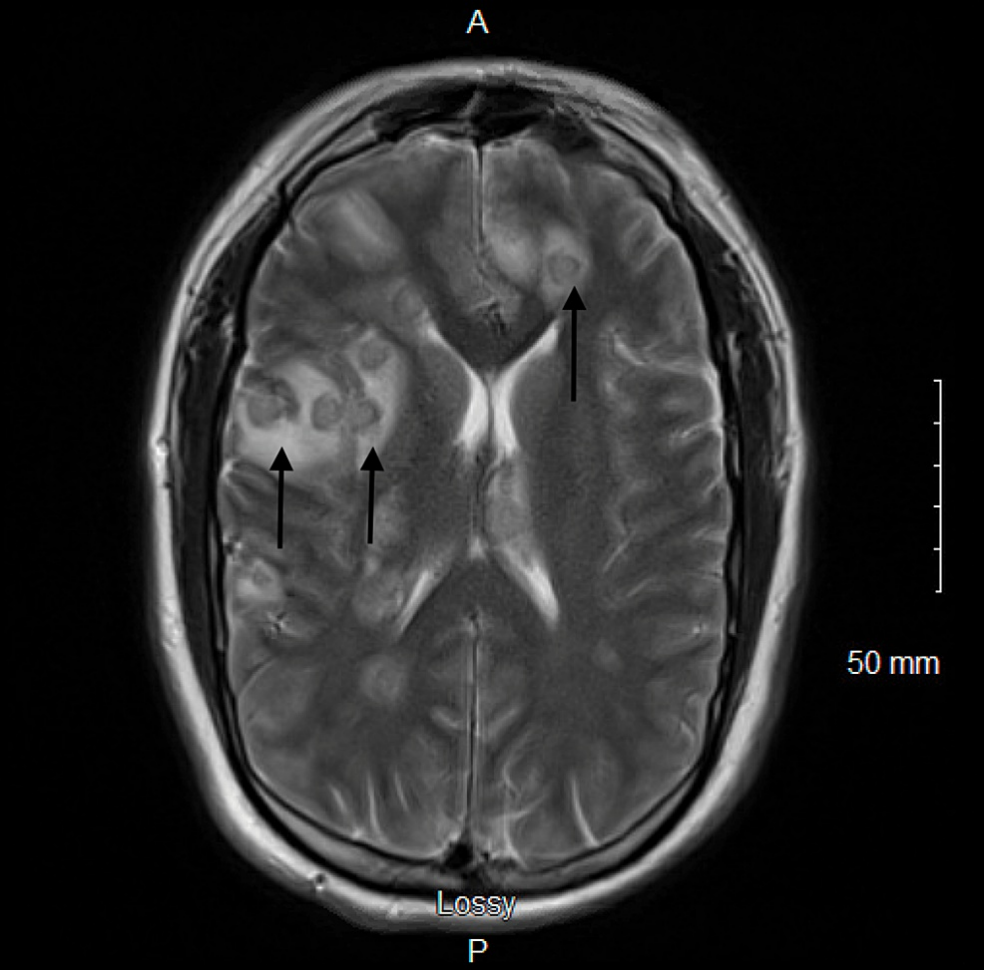
T2-weighted magnetic resonance image sequence of the brain demonstrating multifocal foci with hypointense thin rims around the abscesses and surrounding high-signal-intensity edema.

**Figure 2:**
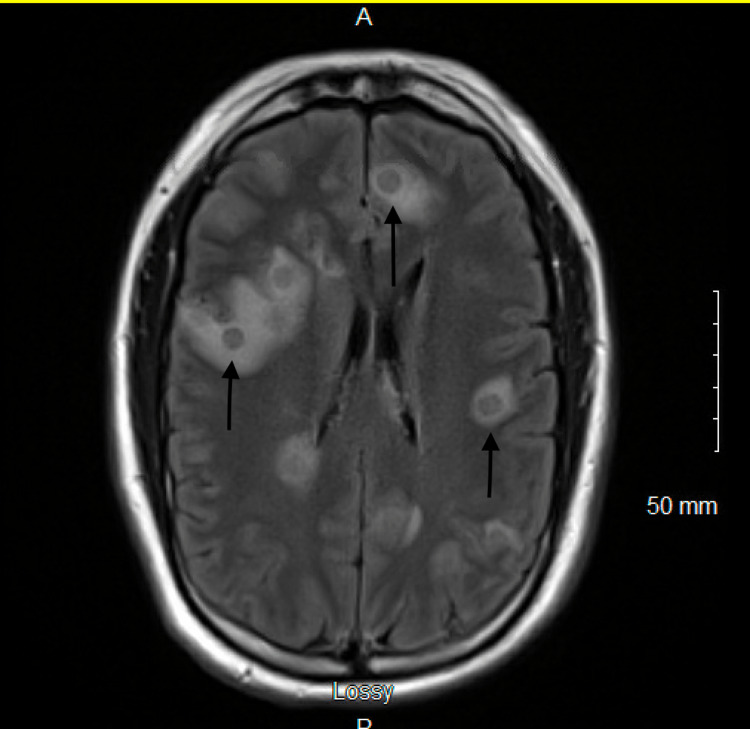
Fluid-attenuated inversion recovery (FLAIR) magnetic resonance image sequence demonstrating multifocal foci with hypointense thin rims and surrounding vasogenic edema located in the supratentorial region of the brain.

His vital signs on admission were the following: blood pressure of 157/87 mmHg, pulse of 79 beats per minute (bpm), and oxygen saturation of 95% on room air. His general appearance was significant for lethargy. On neurologic examination, the patient opened his eyes to verbal stimulus. He presented with a right-sided gaze preference. He was able to follow commands. There was mild left-sided weakness affecting the left upper extremity grip and left lower extremity strength. The patient withdrew to noxious stimuli in all four extremities; however, this was decreased in the left lower extremity. Reflexes were 2+ in the upper and lower extremities bilaterally. Head and neck examination was negative for oral lesions, lymphadenopathy, or neck rigidity. Pulmonary examination revealed normal breath sounds bilaterally. Cardiovascular examination was negative for a systolic, diastolic murmur or abnormal heart rhythm. Abdominal examination was within normal limits, without abdominal tenderness or abnormal bowel sounds. On skin examination, there was no evidence of Janeway lesions, Osler nodes, splinter hemorrhages, or other signs of infective endocarditis. His laboratory results were significant for leukocytosis of 18.1 K cells/µL. Computed tomography (CT) scan of the head showed areas of widespread edema within both the right and left sides of the brain. These findings were described as nonspecific by CT, with a differential diagnosis including widespread areas of abscesses and encephalitis. Further characterization with magnetic resonance imaging (MRI) of the brain with and without contrast reports widespread areas of signal change within the brain including the supratentorial and posterior fossa. The findings were described as areas of restricted diffusion with ring enhancement surrounding the areas of vasogenic edema (Figures 1-2). Given the concern for multiple brain abscesses and with vasogenic edema, he was started on vancomycin, ceftriaxone, and a tapered dose regimen of dexamethasone. Seizure prophylaxis with levetiracetam was also initiated. He was subsequently admitted to the intensive care unit (ICU) for close monitoring of his neurologic status. Neurosurgery was consulted. Infectious disease was consulted.

Upon evaluation by neurosurgery, a course of monitoring on dexamethasone therapy was recommended prior to any neurosurgical intervention at that time. Due to concern for brain abscess, external ventricular drain placement posed a risk of seeding infection into ventricles. A lumbar puncture was recommended. Electroencephalogram was obtained; the evidence of underlying cortical dysfunction predominantly toward the right cortex with moderate diffuse encephalopathy was reported. Upon evaluation by infectious disease, antibiotic therapy regimen with ceftriaxone and vancomycin was continued. Metronidazole and intravenous (IV) trimethoprim-sulfamethoxazole were added to the antibiotic regimen as empiric coverage for possible pathogens causing brain abscesses. Lumbar puncture was subsequently obtained. Cerebrospinal fluid (CSF) analysis revealed an elevated opening pressure (54 mmHg), colorless CSF with a nucleated cell count of 13 cells/µL, 16 red blood cells/µL, a glucose level within normal limits at 74 mg/dl, and an elevated CSF protein level of 94 mg/dl. CSF culture reported no growth and was negative for trophozoites on examination of centrifuged CSF wet mount. CSF meningitis/encephalitis polymerase chain reaction (PCR) panel was negative for the following pathogens: *Haemophilus influenzae*, *Listeria monocytogenes*, *Neisseria meningitidis*, *Streptococcus pneumoniae*, *Cytomegalovirus*, *Enterovirus*, herpes simplex virus 1 and 2, human herpesvirus 6, human parechovirus, varicella zoster virus, and *Cryptococcus neoformans/gattii*.

Serologic studies for *Toxoplasma*, *Histoplasma*, syphilis, *Coccidioides*, tuberculosis, and HIV were negative. On his third intrahospital day, one of the four blood cultures obtained on admission grew pan-sensitive *Streptococcus intermedius*. Antibiotic therapy with trimethoprim-sulfamethoxazole and vancomycin was subsequently discontinued. A transthoracic echocardiogram was negative for signs of vegetations or valvular involvement. Despite being on antibiotic therapy, he had an acute decline in his mental status with associated left-sided weakness, which necessitated endotracheal intubation on his fourth intrahospital day. He underwent flexible fiberoptic bronchoscopy of the right and left lung with bronchoalveolar lavage obtained to identify the possible causes of his worsening clinical status. Bronchoalveolar lavage samples were sent for aerobic, fungal, and viral culture, which reported no growth after finalization. *Aspergillus* galactomannan antigen was negative on bronchoalveolar lavage analysis.

On his seventh intrahospital day, a stereotactic brain biopsy with aspiration of contents from a right frontal brain collection was obtained. Histopathology was consistent with gliosis and acutely inflamed debris consistent with abscess. Bacterial cultures of the aspiration grew *Streptococcus intermedius*, sensitive to ceftriaxone, clindamycin, erythromycin, levofloxacin, penicillin, and vancomycin. Fungal and acid-fast bacilli (AFB) cultures were negative.

After four days of remaining on orotracheal intubation, the patient was successfully extubated on his eighth intrahospital day. The patient subsequently improved clinically. He was discharged with a course of ceftriaxone 2 g IV every 12 (Q12) hours and metronidazole 500 mg by mouth (PO) twice a day (BID) to complete a total duration of six weeks of antibiotic therapy with weekly complete metabolic panel and complete blood count. The patient tolerated the IV course of antibiotic therapy well. Follow-up neuroimaging with brain MRI was obtained, which showed a decrease in the size of previously noted brain abscesses. Neurologic deficits at the time of outpatient follow-up had resolved.

## Discussion

Brain abscess can be secondary to direct spread or hematogenous seeding of infection. While hematogenous spread commonly leads to multiple lesions, multifocal brain abscesses secondary to *Streptococcus intermedius* are rare presentations [4]. While *Streptococcus* and *Staphylococcus* spp. are the most common bacteria cultured from patients with pyogenic brain abscess, *Streptococcus* is mostly identified in brain abscess due to the contiguous spread of infection [3]. *Streptococcus* spp. in brain abscess have been described to be isolated in 30%-60% of cases [5].

The *Streptococcus anginosus* group organisms (*S. anginosus*, *Streptococcus intermedius*, and *Streptococcus constellatus*) are a subgroup of viridans streptococci, which are also referred to as the *Streptococcus milleri* group. These species of streptococcus are gram-positive, catalase-negative facultative anaerobic cocci that are found as normal flora in the upper respiratory, digestive, and reproductive tracts [6]. While this species of streptococci can be found as normal flora in the host, they can at times lead to invasive illness. This was demonstrated in our patient described with multifocal brain abscesses. An abscess is a prominent feature of infection with this group of streptococci; prompt identification and subsequent drainage are the basis of the effective management of infection with this group. Bacteremia may or may not be present at the time of abscess identification in patients with infection secondary to hematogenous seeding, which may make the diagnosis complex.

Due to the infrequent presentation of multifocal brain abscess without direct extension of infection secondary to *Streptococcus intermedius*, this case identifies that pathogens that have classically been described as causing brain abscess by continuation of peri-cranial infection can also be a cause of multifocal lesions secondary to the hematogenous spread of infection.

This case additionally underlines the importance of the multidisciplinary management of the patient with brain abscesses, including the extensive medical management that takes place. Magnetic resonance imaging is the imaging modality of choice in the evaluation of a suspected brain abscess [5]. Computed tomography (CT) in patients with suspected brain abscess has led to the ability to perform stereotactic CT-guided aspiration, as was performed in the presented patient. Stereotactic CT-guided aspiration facilitates diagnoses, and with the use of Gram stain and aerobic, anaerobic, fungal, and acid-fast bacilli (AFB) cultures, microbiologic diagnosis can be made, and guidance for antimicrobial therapy is obtained.

The antimicrobial treatment of bacterial brain abscess is case dependent, and comorbidities and risk factors should be considered. Due to the common bacteria isolated from bacterial brain abscess being streptococci, high-dose intravenous penicillin G or a third-generation cephalosporin (ceftriaxone or cefotaxime) should be included in the initial empiric regimen [5]. While penicillin G has activity against anaerobic species, it lacks coverage against *Bacteroides fragilis*; therefore, the addition of metronidazole should be included when infection with this organism is suspected. As *Staphylococcus aureus* is also a common causal organism in pyogenic brain abscess, vancomycin should also be added to the initial empiric regimen when suspected as a cause of infection, as an increase in community-acquired methicillin-resistant organisms has been described in recent years [5]. Therefore, an initial empiric antimicrobial regimen for suspected bacterial brain abscess often consists of vancomycin, metronidazole, and a third-generation cephalosporin, as was used in the above-described patient. Antimicrobial therapy can subsequently be narrowed upon microbiologic identification. The duration of antimicrobial therapy has classically been 6-8 weeks, with further therapy dependent upon follow-up neuroimaging and the initial surgical management of brain abscesses. Consultation with neurosurgery is most important to determine candidacy for the drainage of abscesses upon presentation.

While immunosuppression leads to an increased risk of brain abscess, this patient was not immunosuppressed. This patient brings to light the importance of the consideration of rare pathogens in the differential diagnosis, such as *Naegleria fowleri*, in patients with history of exposure to freshwater and warm water sources.

## Conclusions

Brain abscess is a life-threatening, focal infection within the brain parenchyma. The infection can occur secondary to direct or hematogenous spread. Otorhinolaryngeal infections causing brain abscess usually lead to solitary brain abscess. While aerobic Streptococcus spp. are frequently identified as a cause of solitary brain abscess, the presentation of multifocal brain abscesses should also lead to the consideration of *Streptococcus intermedius* as a possible cause of infection.
